# Increased MCL1 dependency leads to new applications of BH3-mimetics in drug-resistant neuroblastoma

**DOI:** 10.1038/s41416-023-02430-8

**Published:** 2023-09-19

**Authors:** Maureen Jacob, Sara Wiedemann, Daniela Brücher, Nadja M. Pieper, Moni Birkhold, Vinzenz Särchen, Jan Jeroch, Melanie C. Demes, Steffen Gretser, Yannick Braun, Elise Gradhand, Florian Rothweiler, Martin Michaelis, Jindrich Cinatl, Meike Vogler

**Affiliations:** 1 Institute for Experimental Cancer Research in Pediatrics, Goethe-University Frankfurt, Frankfurt am Main, Germany; 2 Dr. Senckenberg Institute of Pathology, University Hospital Frankfurt, Frankfurt am Main, Germany; 3 Department of Pediatric and Perinatal Pathology, Dr. Senckenberg Institute of Pathology, Goethe-University Frankfurt, Frankfurt am Main, Germany; 4 Department of Pediatric Surgery and Pediatric Urology, University Hospital Frankfurt, Goethe-University Frankfurt, Frankfurt am Main, Germany; 5 Institute for Medical Virology, Goethe-University Frankfurt, Frankfurt am Main, Germany; 6 Dr. Petra Joh-Forschungshaus, Frankfurt am Main, Germany; 7 School of Biosciences, University of Kent, Canterbury, UK; 8 German Cancer Consortium (DKTK) Partner Site Frankfurt/Mainz, Frankfurt am Main, Germany

**Keywords:** Cancer therapeutic resistance, Paediatric cancer, Cancer immunotherapy

## Abstract

**Background:**

Neuroblastoma is a paediatric cancer that is characterised by poor prognosis for chemoresistant disease, highlighting the need for better treatment options. Here, we asked whether BH3-mimetics inhibiting BCL2 proteins may eliminate chemoresistant neuroblastoma cells.

**Methods:**

We utilised cisplatin-adapted neuroblastoma cell lines as well as patient tissues before and after relapse to study alterations of BCL2 proteins upon chemoresistance.

**Results:**

In a direct comparison of cisplatin-resistant cells we identified a prominent loss of sensitivity to BCL2/BCL-X_L_ inhibitors that is associated with an increase in MCL1 dependency and high expression of MCL1 in patient tumour tissues. Screening of FDA-approved anti-cancer drugs in chemoresistant cells identified therapeutics that may be beneficial in combination with the clinically tested BH3-mimetic ABT263, but no synergistic drug interactions with the selective MCL1 inhibitor S63845. Further exploration of potential treatment options for chemoresistant neuroblastoma identified immunotherapy based on NK cells as highly promising, since NK cells are able to efficiently kill both parental and chemoresistant cells.

**Conclusions:**

These data highlight that the application of BH3-mimetics may differ between first line treatment and relapsed disease. Combination of NK cell-based immunotherapy with BH3-mimetics may further increase killing of chemoresistant neuroblastoma, outlining a new treatment strategy for relapsed neuroblastoma.

## Introduction

Neuroblastoma is a paediatric cancer that develops from immature neural-derived cells and is frequently located in the adrenal glands, upper abdomen or chest of the patients. Although it is the most common extracranial solid tumour in children, the overall incidence of neuroblastoma remains low, with ~10 in a million. With a median age of less than 2 years, the affected children are typically very young [1]. Neuroblastoma has a highly heterogeneous outcome variability, with children less than a year old having better outcomes than older children. While some children are cured from the disease or even display spontaneous regressions, the 5-year survival is only around 40% in the high-risk group, highlighting the urgent need for better treatment options in this patient group [2].

One potential targeted treatment avenue for high-risk neuroblastoma may involve the induction of apoptosis by selective inhibition of the anti-apoptotic B-cell lymphoma 2 (BCL2) proteins. The anti-apoptotic BCL2 proteins are located at the outer mitochondrial membrane and prevent the release of cytochrome c into the cytosol. On a molecular level, the anti-apoptotic BCL2 proteins sequester and inhibit the pro-apoptotic BCL2 proteins via their BCL2 homology 3 (BH3) domain. This balance between the anti- and pro-apoptotic BCL2 family proteins may be shifted in favour of apoptosis by using small-molecule BH3-mimetics, pharmacologically active compounds that functionally resemble BH3-only proteins and occupy the same binding interface on the anti-apoptotic BCL2 proteins. In some cancer types, treatment with BH3-mimetics may be sufficient to induce apoptosis, and with the development of ABT199/venetoclax, the first BH3-mimetic has obtained approval for the treatment of leukaemia [3, 4]. The clinical success of ABT199 and its ability to effectively cure leukaemia in many patients has highlighted the potential of BH3-mimetics as mechanism-based anti-cancer therapeutics, and multiple studies are currently ongoing to expand the use of BH3-mimetics to other malignancies including solid tumours [5].

We and others have recently shown that BH3-mimetics may also be highly promising in the treatment of neuroblastoma [6–9]. Besides the inhibition of BCL2 with ABT199, also the inhibition of BCL-X_L_ or MCL1 with their selective antagonists A1331852 [10] or S63845 [11] is able to induce apoptosis in multiple neuroblastoma cells including primary cells [6, 8]. Thereby, the selective BH3-mimetics are able to displace the pro-apoptotic binding partners BIM and BAK, leading to the activation and oligomerisation of BAX and BAK and subsequently to the induction of mitochondrial apoptosis [6].

In this study, we asked whether BH3-mimetics may also be effective at inducing apoptosis in chemoresistant neuroblastoma cells. Intensive chemotherapy based on cisplatin and other cytotoxic drugs like vincristine and etoposide is the standard induction treatment for most neuroblastoma patients. However, patients in the high-risk group often relapse, and relapsed disease coincides with the occurrence of drug-resistant cells that display cross-resistance to other agents and fail to respond to second-line chemotherapy treatment. These drug-resistant cells have multiple genetic and epigenetic alterations that affect metabolism, cytoskeleton and also the presentation of surface antigens. These alterations induce drug resistance independently of drug transports, as an increased malignant behaviour in neuroblastoma cells with acquired multi-drug resistance has been observed independently of P-glycoprotein expression [12]. Clinically, chemoresistant disease is a major barrier that needs to be overcome in order to improve the overall survival of children with neuroblastoma. One currently applied strategy is the combination of second-line chemotherapy with targeted agents, and addition of an antibody against the disialoganglioside GD2 has been shown to improve second-line chemotherapy, highlighting the potential of targeted agents in this setting [13, 14].

Recent transcriptional profiling of single cells and lineage profiling has revealed that two different cell entities with different epigenetic programming and differentiation status may lead to the formation of neuroblastoma, namely the committed adrenergic (ADRN) and the undifferentiated mesenchymal (MES) cells [15, 16]. Whereas the MES type is derived from neural crest-like cells, the ADRN type is already committed to the adrenergic cell type. However, cells can switch between the two types, highlighting the transcriptional plasticity of neuroblastoma. Of note, chemotherapy may select for the less differentiated MES type [17], thus supporting the earlier observations that drug resistance may be associated with altered epithelial-to-mesenchymal transition.

Here, we investigated whether BH3-mimetics may be able to overcome resistance to cisplatin in neuroblastoma. To this end, we utilised neuroblastoma cell lines that have adapted to the presence of cisplatin by continuous culture in increasing concentrations of cisplatin. These cisplatin-resistant cells display reduced NCAM expression mediating increased invasion [18] and elevated tumour forming potential in vivo [19]. Gene expression analysis confirmed a global shift associated with pro-angiogenic activity [20].

Using these cells as models for drug-resistant cells, we observed a marked shift in the response to BH3-mimetics. More precisely, the response to BCL2 and BCL-X_L_ inhibitors was reduced in cisplatin-resistant cells, which coincided with an increased dependency on MCL1 and hence increased sensitivity to selective MCL1 inhibitors. By aiming to identify therapeutic options that can be combined with BH3-mimetics to overcome cisplatin resistance, we identified cellular immunotherapy rather than FDA-approved drugs as highly promising approach combined with MCL1 inhibitors. To this end, we and others recently discovered that BH3-mimetics may act together with Natural Killer (NK) cell-based immunotherapy to promote tumour cell killing [21, 22]. The importance of the immune system in controlling neuroblastoma is demonstrated by the improved outcome of tandem transplants versus single autologous stem cell transplant post chemotherapy for high-risk neuroblastoma [23, 24]. In this setting, also NK cell-based therapy may be given, and several clinical trials are ongoing. Thereby, oncogenic expression of MYC has been shown to dampen the expression of activating NK cell receptors in high-risk neuroblastoma, indicating that MYC expression may serve as a biomarker for NK cell therapy [25]. While BH3-mimetics may not act synergistically with chemotherapy to overcome drug resistance, we observed a beneficial response when combined with cellular immunotherapy.

## Materials and methods

### Chemicals

The BH3-mimetics ABT199, ABT263, A1331852, S63845, S64135 and AZD-5591 (all from Selleck, Houston, TX) were diluted in DMSO and used at the indicated concentrations. To stain spheroids for microscopy, HOECHST33342 and propidium iodide were used (Sigma-Aldrich, Taufkirchen, Germany). The translation inhibitor cycloheximide (CHX) was obtained from Sigma-Aldrich. To identify synergistic drug combinations, the anti-cancer approved drug library (TargetMol, Wellesley Hills, MA) containing 397 compounds diluted in DMSO was used. For drug screening, these were diluted in 96-well plates and used at 0.03, 0.3, 3, and 30 μM concentrations.

### Cell culture

CHLA15 and CHLA20 cells were kindly provided by the Children’s Oncology Group Cell Culture Repository. The neuroblastoma cell lines IMR32 (DSMZ, Braunschweig, Germany), LAN5 (DSMZ, Braunschweig, Germany), NLF (Prof. Dr. Angelika Eggert, Charite Berlin, Germany), and SH-SY5Y (ATCC, Manassas, VA, US) were cultured in RPMI-1640 GlutaMAX-I (Life Technologies, Eggenstein, Germany) supplemented with 10% or 20% (for LAN5) fetal calf serum (Life Technologies), 100 IU/ml penicillin, and 100 µg/ml streptomycin (Life Technologies).

The cisplatin-resistant sublines IMR32^r^CDDP^1000^ (adapted to growth in the presence of cisplatin 1000 ng/ml), LAN5^r^CDDP^1000^ (adapted to cisplatin 1000 ng/ml), NLF^r^CDDP^500^ (adapted to cisplatin 500 ng/ml), and SH-SY5Y^r^CDDP^1000^ (adapted to cisplatin 1000 ng/ml) were established by continuous exposure to stepwise increasing drug concentrations as previously described [26] and derived from the resistant cancer cell line (RCCL) collection (https://research.kent.ac.uk/industrial-biotechnology-centre/the-resistant-cancer-cell-line-rccl-collection/) [27]. To simplify the nomenclature of the resistant cells, these are named CDDP throughout the manuscript. For the CDDP cells, 0.5–1 μg/ml of cisplatin (Teva GmbH, Ulm, Germany) was added to the culture, and experiments were performed after cisplatin washout for 7 days. All cell lines were routinely monitored for mycoplasma contamination. For spheroid formation, 5000 cells were added to U-bottom 96-well plates coated with anti-adherence solution (STEMCELL Technologies, Cologne, Germany) followed by centrifugation at 1000 × *g* for 10 min. Spheroids were allowed to grow for 3 days before addition of drugs or NK cells. NK cells were isolated from peripheral blood mononuclear cells (PBMCs) derived from healthy donors (DRK Blutspendedienst, Frankfurt, Germany) by density gradient centrifugation with Histopaque-1077 (Sigma-Aldrich) followed by enrichment with Human NK Cell Enrichment kit (STEMCELL Technologies). To expand and activate NK cells, isolated NK cells were cultured for 11 days in NK-MACS medium (Miltenyi Biotec, Bergisch Gladbach, Germany) and 10 ng/ml human IL-15 (Peprotech, Rocky Hill, CT) before experiments. Purity and activation of NK cells were routinely controlled by staining of CD56/CD16 and flow cytometry at FACS Canto II (BD Biosciences, Heidelberg, Germany) as described previously [22].

### Analysis of viability and cell death

To quantify neuroblastoma cell viability the CellTiterGlo (CTG) assay (Promega) was used. Briefly, cells were seeded at 0.5 × 10^5^/cm^2^ in white plates before drug treatment. Viability was assessed using luminescence measured at Tecan Infinite M200 plate reader. Loss of mitochondrial membrane potential (MMP) was assessed by staining with 50 nM of tetramethylrhodamine-methylester (TMRM) and flow cytometry. Microscopy was performed on spheroids using the ImageXpress XLS Widefield analysis system (Molecular Devices, Sunnydale, CA). The best projection image of a z-stack was stored and analysed using MetaXpress (Molecular Devices, v 6.5.4.532). To detect active caspases, the CellEvent® Caspase-3/7 Green Detection Reagent (Life Technologies) was used according to the manufacturer’s instruction.

### Protein lysis, Western blotting and immunoprecipitation

For analysis of protein expression, cells were lysed in 0.5% TritonX buffer. Western blotting was performed using the following antibodies: mouse anti-BCL2 (Dako, M088701-2), rabbit anti-BCL-X_L_ (Cell Signaling, 2762S), rabbit anti-MCL1 (Enzo, ADI-AAP-240F), rabbit anti-BIM (Cell Signaling, 3183S), mouse anti-NOXA (Enzo, ALX-804-408), mouse anti-BAX (BD Bioscience, 610983), mouse anti-GAPDH (BioTrend, 5G4-6C5) or mouse anti-VINCULIN (Sigma-Aldrich, Germany, Cat. No. V9131-100UL). For detection, goat anti-rabbit or goat anti-mouse IgG conjugated to horseradish peroxidase (Santa Cruz Biotechnology, Santa Cruz, CA, USA; Cat. No. SC-2004, SC-2005) and enhanced chemiluminescence (Amersham Biosciences, Freiburg, Germany) were used. For immunoprecipitation (IP) of BIM, anti-BIM antibody (Cell Signalling, 2819S) was crosslinked with Protein G Dynabeads (Thermo Scientific) using Dimethyl pimelimidate. IP and subsequent washes were performed in Tris lysis buffer containing 1% CHAPS.

### RNA isolation and qRT-PCR

Total cellular RNA was isolated from cultured cells using the peqGOLD MicroSpin total RNA kit and peqGOLD total RNA kit including DNase I digestion (PeqLab, Erlangen, Germany) or from FFPE sections using Maxwell® RSC RNA FFPE Kit (Promega). For cDNA synthesis mRNA (1 µg) was used for RevertAid first strand cDNA synthesis kit (Thermo Fisher, Roskilde, Denmark). qRT-PCR was performed on a QuantStudio™ 7 Flex system (Applied Biosystems, Darmstadt, Germany) using Sybr™ Green PCR master mix (Applied Biosystems) and following primers: MCL1 (AAGCCAATGGGCAGGTCT, TGTCCAGTTTTCCGAAGCAT), G6PD (ATCGACCACTACCTGGGCAA, TTCTGCATCACGTCCCGGA), RPII (GCACCACGTCCAATGACAT, GTGCGGCTGCTTCCATAA).

### siRNA knockdown

For knockdown of MCL1 the parental or CDDP SH-SY5Y cells were electroporated using Neon transfection system (Thermo Fisher) with two pulses of 20 ms at 1200 V and 50 nM of silencer select siRNAs (#s8583, #s8585) (Thermo Fisher). Non-silencing siRNA was used as control. Immediately after electroporation, cells were seeded in 96-well white plates for analysis of viability using CTG assay or in 6- well plates for protein lysis at 48 h after electroporation.

### Immunohistochemistry (IHC)

Neuroblastoma tumour tissues from patients at initial diagnosis (primary tumour) and at relapse (metastasis) were stained using anti-MCL1 antibody (Abcam ab32087). Local ethical approval (Study SPO-04-2015) and patient consent was obtained through the UCT Biobank.

### Statistics

To quantify drug synergy the Bliss score was calculated using Synergyfinder (https://synergyfinder.fimm.fi/). For this score, values >1 signify synergism, whereas values <−1 signify antagonism and everything in between additivity [28]. Quantification of protein expression was performed using ImageJ 3.1 software. For comparison of two samples calculation of statistical significance with two-tailed, two sample, equal variance Student’s *t* test was done in Excel.

## Results

### Cisplatin resistance is associated with a shift in response to selective BH3-mimetics

Acquired resistance to chemotherapy is a major clinical problem in neuroblastoma, and overcoming this chemoresistance with precision medicine like BH3-mimetics may be a promising approach in relapsed neuroblastoma. To study how acquired resistance to cisplatin in neuroblastoma influences the response to BH3-mimetics, we used a selection of cell lines that were adapted to growth in the presence of cisplatin, namely IMR32, Lan5, NLF and SH-SY5Y parental cell lines and their respective CDDP adapted sub-lines. All sub-lines that were adapted to cisplatin displayed significantly increased resistance to cisplatin (Supplementary Fig. 1), confirming their acquired resistance to cisplatin.

Next, we asked whether the acquired resistance to cisplatin affects the response to BH3-mimetics. To this end, we applied selective antagonists of the main anti-apoptotic BCL2 family proteins BCL2, BCL-X_L_ or MCL1. Parental and CDDP cells were exposed to different concentrations of ABT199, A1331852 or S63845 before analysis of cell viability. As described previously, in particular the parental Lan5 and SH-SY5Y cells displayed marked sensitivity to both ABT199 and A1331852, while the IMR32 and NLF cells were more sensitive to the BCL-X_L_ inhibitor A1331852 than to the BCL2 inhibitor ABT199 [6]. Of note, none of the parental cell lines responded to the selective MCL1 inhibitor S63845.

**Fig. 1:**
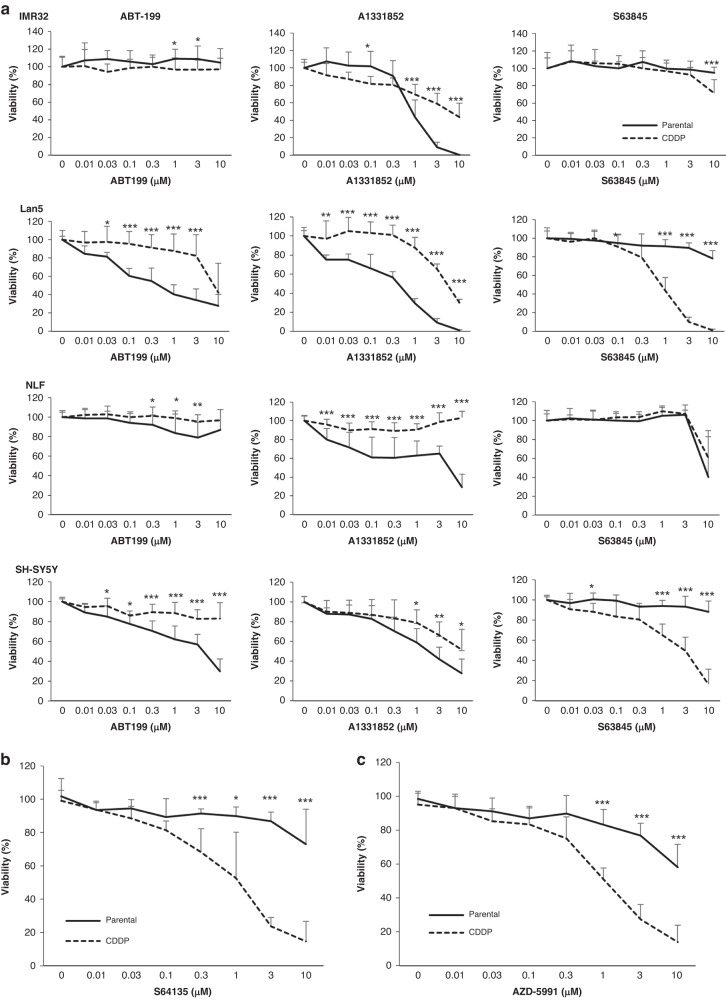
Cisplatin-resistant neuroblastoma cells display a shift in response to BH3-mimetics. **a** IMR-32, Lan5, NLF or SH-SY5Y neuroblastoma cell lines were treated with different concentrations of the BH3-mimetics ABT199, A1331852 or S63845 for 72 h before analysis of viability using CTG assay. The response of parental cells (solid lines) was compared to the response of CDDP-resistant cells (dashed lines). Data shown are mean + standard deviation (SD) with *n* = 3–4. *p < 0.05, **p < 0.01, ***p < 0.001. Parental or CDDP-resistant SH-SY5Y cells were treated with the clinically tested MCL1 inhibitors S64315 (**b**) or AZD-5991 (**c**) for 72 h before analysis of viability using CTG assay. Data shown are mean + SD (*n* = 3), *p < 0.05, **p < 0.01, ***p < 0.001.

In contrast to the parental cells, the CDDP cells displayed reduced sensitivity to both ABT199 and to A1331852. This pattern was observed for all cell lines that displayed sensitivity to the BH3-mimetics as parental cells, indicating that this shift in response may be a shared feature of cisplatin resistance. Surprisingly, the CDDP Lan5 and SH-SY5Y became significantly more sensitive to the MCL1 inhibitor S63845, where viability was now reduced at submicromolar concentrations (Fig. 1a).

Next, we asked whether this increased sensitivity towards the MCL1 inhibitor S63845 that was observed in some of the CDDP cell lines could also be extended to MCL1 inhibitors currently tested in clinical trials. To this end, we investigated the effect of AZD-5991 or S64315/MIK665 in the parental or CDDP SH-SY5Y cells. In line with the effect observed for S63845, loss of viability induced by AZD-5991 or S64315 was significantly higher in the CDDP-resistant cells as compared to the parental cells (Fig. 1b, c).

### Altered response to BH3-mimetics is maintained in 3D spheroids

Since the culture of tumour cells in monolayer culture may not faithfully model the drug response in 3D tumours, and since chemoresistance in neuroblastoma has been shown to alter the adhesion properties of neuroblastoma cells, we extended our study to 3D spheroid models. Spheroids were established from selected cell lines (IMR32 and SH-SY5Y) without additional scaffolding components in ultra-low attachment plates as described previously [22]. Both parental and CDDP cells formed compact spheroids that, to some extent, differed in their shape and morphology (Supplementary Fig. 2). To investigate how the tumour spheroids responded to BH3-mimetics, established spheroids were exposed to ABT199, A1331852 or S63845. In both IMR32 and SH-SY5Y, the response to ABT199 and A1331852 was reduced in the CDDP cells compared to the parental cells, with less cell death induced in the CDDP cells (Supplementary Figs. 2 and 3). In sharp contrast, the MCL1 inhibitor S63845 induced more tumour cell killing in the CDDP cells compared to the parental spheroids. This increased sensitivity of CDDP cells to the MCL1 inhibitor S63845 was confirmed by an analysis of caspase activity based on a fluorescent caspase substrate (Supplementary Fig. 2B). Taken together, these experiments demonstrate that the shift in response to BH3-mimetics was comparable between 2D monolayer and 3D spheroid culture, and that under both culture conditions the resistance to CDDP coincided with a shift from BCL2/BCL-X_L_ dependency towards MCL1 dependency.

**Fig. 2:**
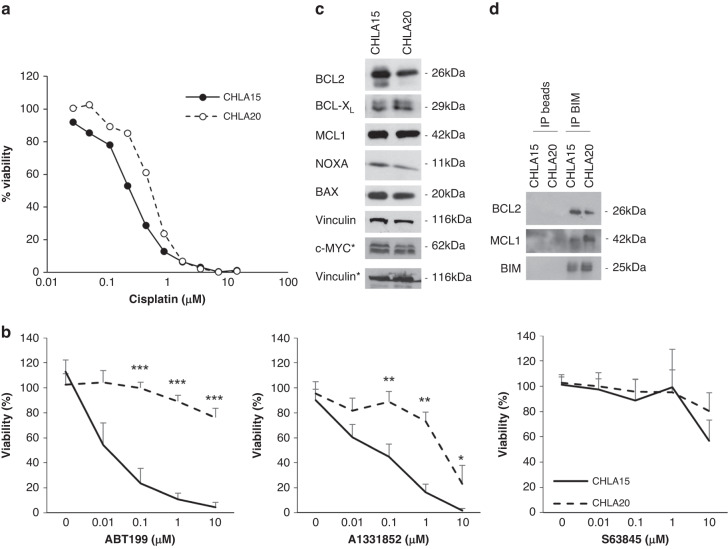
Altered sensitivity to BH3-mimetics occurs in vivo during chemotherapy. **a** CHLA15 or CHLA20 cells were treated with different concentrations of cisplatin for 72 h before analysis of viability using CTG assay. **b** CHLA15 or CHLA20 cells were treated with different concentrations of the BH3-mimetics ABT199, A1331852 or S63845 for 72 h before analysis of viability using CTG assay. **c** Protein expression of CHLA15 and CHLA20 cells was analysed by Western blotting. One representative blot out of three independent experiments is shown. **d** IP of BIM was performed in CHLA15 and CHLA20 cells with ProtG dynabeads serving as control. Interaction of BIM with BCL2 and MCL1 was investigated by Western blotting.

To investigate whether the phenotype observed in our cell culture models may reflect the development of chemoresistance in vivo during the treatment of neuroblastoma patients with chemotherapy, we utilised a cell line model that was derived from a neuroblastoma patient at diagnosis (CHLA15) and at relapse after cisplatin-based chemotherapy (CHLA20). Treatment of the CHLA15 and CHLA20 cells with cisplatin confirmed that the CHLA20 cells display a reduced response to cisplatin (Fig. 2a). Comparison of the response to BH3-mimetics in the CHLA15 and CHLA20 cells demonstrates a marked and highly significant loss of sensitivity to BCL2 and BCL-X_L_ inhibition, as evidenced by the resistance to ABT199 and A1331852 in the CHLA20 cells (Fig. 2b). The expression of BCL2 proteins was not altered in these cells with the exception of BCL2, which was downregulated in the CHLA20 cells (Fig. 2c). To investigate whether the difference in response to BH3-mimetics may be explainable by different complexes formed within the BCL2 protein family, we performed IP of BIM and investigated its interaction with the anti-apoptotic proteins BCL2 and MCL1. In comparison to the CHLA15 cells, in the resistant CHLA20 cells BIM was less associated with BCL2 and more in complex with MCL1 (Fig. 2d). These data indicate that the different response to BH3-mimetics may be associated with different complex formations and altered priming of the resistant cells.

### Resistance to cisplatin results in altered apoptosis priming and mitochondrial apoptosis

**Fig. 3:**
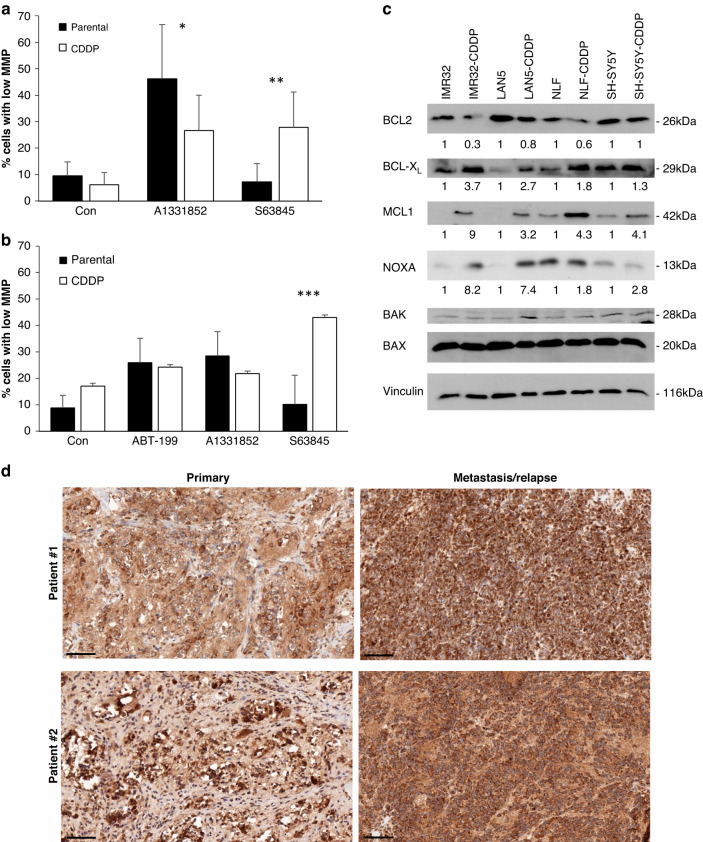
Shifted response to BH3-mimetics is associated with altered apoptotic signalling. IMR-32 (**a**) or Lan5 (**b**) cells were treated with ABT199, A1331852 or S63845 (1 μM) for 24 h before analysis of MMP using staining with TMRM and flow cytometry. Data shown are mean + SD (*n* = 4). Statistical analysis was done using *t*-test (*p < 0.05, **p < 0.01, ***p < 0.001). **c** Protein expression of parental and CDDP-resistant cells was analysed by Western blotting. One representative blot out of three independent experiments is shown. All experiments were quantified by densitometry and the average protein expression is indicated below each blot. **d** Neuroblastoma tumour tissues obtained from two individual patients at initial diagnosis (primary) or relapse (metastasis/relapse) was stained with anti-MCL1 antibody. Brown colour indicates positive MCL1 staining. Scale bar represents 100 μm.

To confirm that this shift from sensitivity to BCL-X_L_ inhibition towards sensitivity to MCL1 inhibition is mediated by the mitochondrial apoptosis pathway, we analysed the loss of MMP in selected cell lines. These data demonstrate that, as expected, the apoptotic signalling is altered upstream of mitochondrial permeabilisation in the cisplatin-resistant cells (Fig. 3a, b). The loss of MMP is regulated by the interactions of pro- and anti-apoptotic BCL2 proteins that are altered by the BH3-mimetics investigated here. Therefore, we asked whether the resistance to CDDP influences the expression of BCL2 proteins. In line with the observations in the CHLA20 cells, the expression of BCL2 is reduced in some of the CDDP cells. In addition, an increase in BCL-X_L_ and MCL1 expression was observed in the CDDP cells, indicating that these proteins may become upregulated during the acquisition of CDDP resistance. Alongside the anti-apoptotic proteins, the main pro-apoptotic binding partner of MCL1, the BH3-only protein NOXA, was increased in some of the cell lines investigated here as well (Fig. 3c).

### Resistance to cisplatin is associated with increased MCL1 expression and MCL1 dependency

**Fig. 4:**
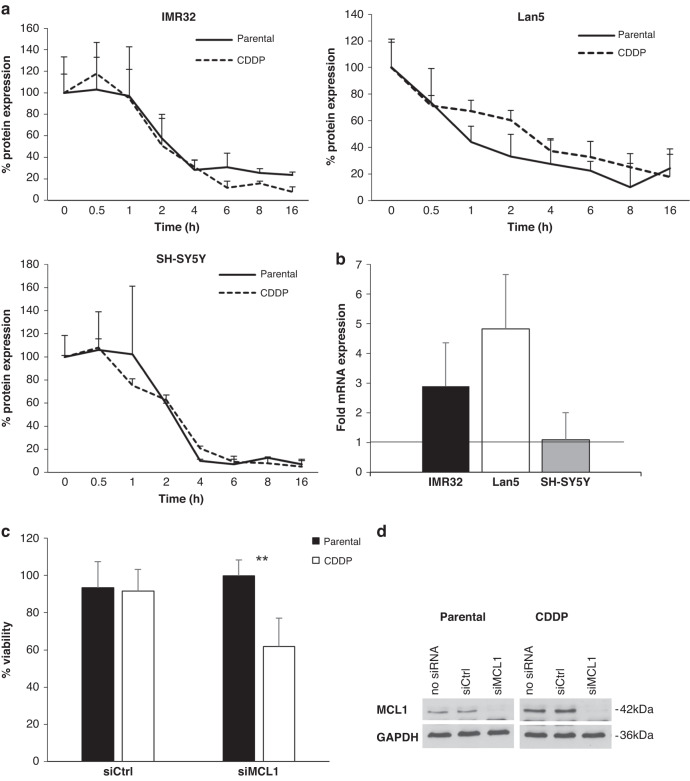
Increased dependency on MCL1 in cisplatin-resistant neuroblastoma cells. **a** MCL1 protein stability was assessed by CHX chase experiment in IMR32, Lan5 or SH-SY5Y cells. Expression of MCL1 was analysed at different time points after addition of CHX (1 μg/ml) by Western blotting and normalised to GAPDH loading control in parental or CDDP cells. Quantification of protein expression was done for three independent experiments. Data shown are mean + SD (*n* = 3). **b** MCL1 mRNA expression was analysed by qRT-PCR in the IMR32, Lan5 and SH-SY5Y cells with RPII and G6PD serving as housekeeping genes. Data shown are the mean of the relative expression of MCL1 in the CDDP cells normalised to parental control cells + SD (*n* = 3). **c**, **d** Knockdown of MCL1 was performed by electroporation with 50 nM of siRNA in parental or CDDP-resistant SH-SY5Y cells. At 48 h after transfection, viability was assessed by CTG assay (**c**) and protein lysis was performed for Western blotting (**d**).

Since cell lines do not always recapitulate the alterations occurring in patients, we extended our studies to neuroblastoma sections of FFPE samples from patients. Using paired tissues from patients at primary diagnosis and after relapse, we investigated the expression of MCL1 using IHC. We observed high nuclear and cytoplasmic expression of MCL1 in all tumour tissues examined, with more homogeneously high expression of MCL1 in the metastasis (Fig. 3d). Increased expression of MCL1 was also observed by qRT-PCR analysis on the same tissue sections (Supplementary Fig. 4). MCL1 is highly regulated both on a transcriptional level as well as post-transcriptionally by protein degradation. To investigate whether the upregulation of MCL1 in the CDDP cell lines was mediated by protein stability or by increased transcription, we first investigated the stability of MCL1 in the parental and CDDP cells. CHX chase experiments displayed no significant differences in the stability with comparable half-lives of MCL1 of around 2 h in the parental or CDDP cells, indicating that the elevated expression of MCL1 in the CDDP cells was not due to increased stability (Fig. 4a). To investigate whether the increase in MCL1 was mediated by enhanced transcription of MCL1, we performed qRT-PCR analysis. In the IMR32 and Lan5 cells, the mRNA expression of MCL1 was induced in the CDDP-resistant cells compared to the parental cells (Fig. 4b). Therefore, we conclude that the expression of MCL1 in the CDDP-resistant cells is mediated by increased transcription rather than altered protein stability.

To further investigate the role of MCL1 in CDDP-resistant cells, we reduced the expression of MCL1 using siRNA technology. Western blot analysis confirmed efficient loss of MCL1 in both parental and CDDP cells. Interestingly, knockdown of MCL1 was sufficient to reduce the viability of CDDP cells, while loss of MCL1 did not affect viability of parental SH-SY5Y cells (Fig. 4c, d). These experiments highlight the importance of MCL1 as promising therapeutic target in chemoresistant neuroblastoma.

### Combined inhibition of all main anti-apoptotic BCL2 proteins is sufficient to overcome cisplatin resistance

We previously described that BH3-mimetics could act highly synergistically in multiple types of paediatric cancer including neuroblastoma [8]. To investigate whether BH3-mimetics may also act synergistically in CDDP-resistant cells, we combined the individual selective BH3-mimetics with each other (Supplementary Fig. 5A, B). In line with our previous observations, BH3-mimetics act highly synergistically in the parental IMR32 and SH-SY5Y cells, and this synergy was observed for all combinations of BH3-mimetics, albeit to different extents. In the CDDP-resistant cells, the synergy was maintained for those combinations including the MCL1 inhibitor S63845, whereas the combined inhibition of BCL2 and BCL-X_L_ was less effective and even antagonistic in the CDDP SH-SY5Y cells, thus underscoring the importance of MCL1 as a therapeutic target in chemoresistant neuroblastoma. Since MCL1 inhibition was synergistic with both BCL2 or BCL-X_L_ inhibition in the CDDP-resistant cells, we asked whether the combined inhibition of BCL2 and BCL-X_L_ with the clinically tested compound ABT263 (navitoclax) may also be synergistic with S63845. The combination of ABT263 with S63845 was highly synergistic in both parental and CDDP-resistant SH-SY5Y and IMR32 cells (Supplementary Fig. 6). These data demonstrate that the neutralisation of all main anti-apoptotic BCL2 proteins is sufficient to overcome chemoresistance and to efficiently eliminate all neuroblastoma cells.

### Identification of novel synergistic treatment combinations with BH3-mimetics

However, the combination of BH3-mimetics is currently not a viable treatment option, and hence we asked whether the addition of a single BH3-mimetic to chemotherapy may be sufficient to resensitise resistant neuroblastoma cells. Therefore, we combined the selective BH3-mimetics with different types of clinically applicable chemotherapeutics. However, neither cisplatin nor etoposide or doxorubicin showed substantial synergy with any of the BH3-mimetics in the CDDP cells (Supplementary Fig. 7).

**Fig. 5:**
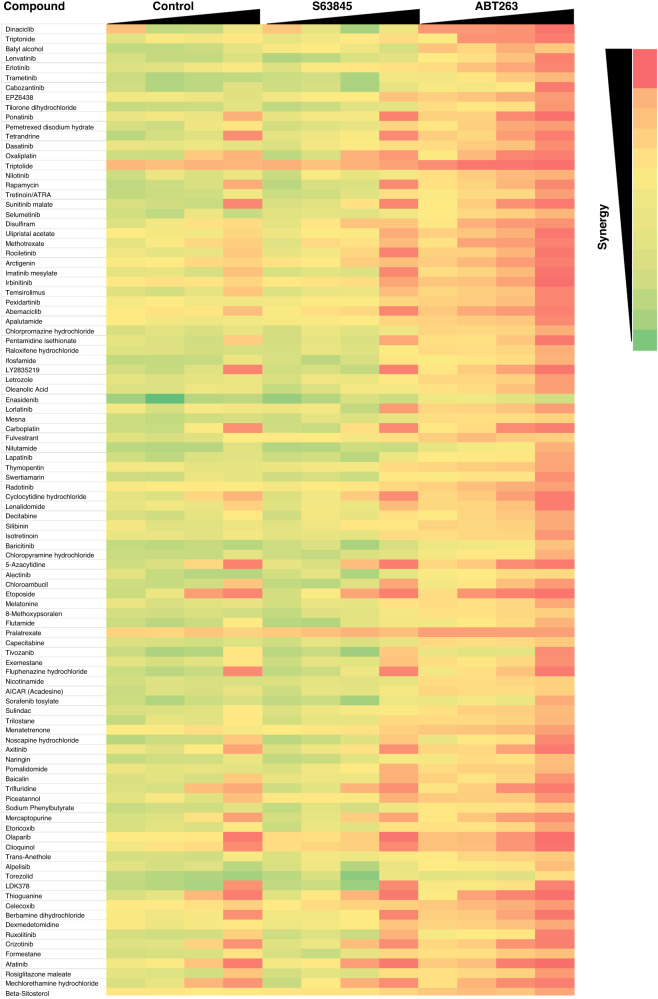
Screen of FDA-approved anti-cancer drugs combined with BH3-mimetics. CHLA-20 cells were exposed to at 0.03, 0.3, 3, or 30 μM of the anti-cancer approved drug library containing 397 compounds either alone (control) or in combination with the MCL1 inhibitor S63845 (1 μM) or the BCL2/BCL-X_L_ inhibitor ABT263 (0.1 μM). Cell viability was assessed at 72 h using CTG assay. The mean effect of 3–5 experiments was calculated and drug efficacy was displayed as colour coding with red showing highest effects. Compounds were sorted by calculating the combined additional effect of ABT-263 across all concentrations, and the 100 most effective compounds are shown.

To identify novel ways to maximise the effect of BH3-mimetics in CDDP neuroblastoma we performed a drug screen with 397 FDA-approved anti-cancer drugs alone and in combination with BH3-mimetics. In order to simplify this screen, we combined the inhibition of BCL2 and BCL-X_L_ using ABT263, which inhibits both BCL2 and BCL-X_L_ [29]. Synergistic interaction with inhibition of MCL1 was investigated using S63845. In order to more faithfully capture the CDDP resistance occurring in patients, we performed this screen in CHLA20 cells that were obtained from a patient at relapse after CDDP therapy. Out of the 397 drugs investigated, none showed convincing synergistic interaction with the MCL1 inhibitor S63845, while many compounds displayed synergistic interaction with ABT263 (Fig. 5). Amongst the compounds that displayed synergy with ABT263 there were multiple kinase inhibitors including the known ABT263 sensitiser trametenib and several CDK inhibitors, e.g. Dinaciclib and Abemaciclib [30]. In addition, two natural compounds applied in traditional Chinese medicine, triptonide and triptolide, displayed synergy with ABT263. This combination was further followed up in both CHLA20 and IMR32-CDDP cells, confirming synergistic drug interaction (Supplementary Fig. 8).

**Fig. 6:**
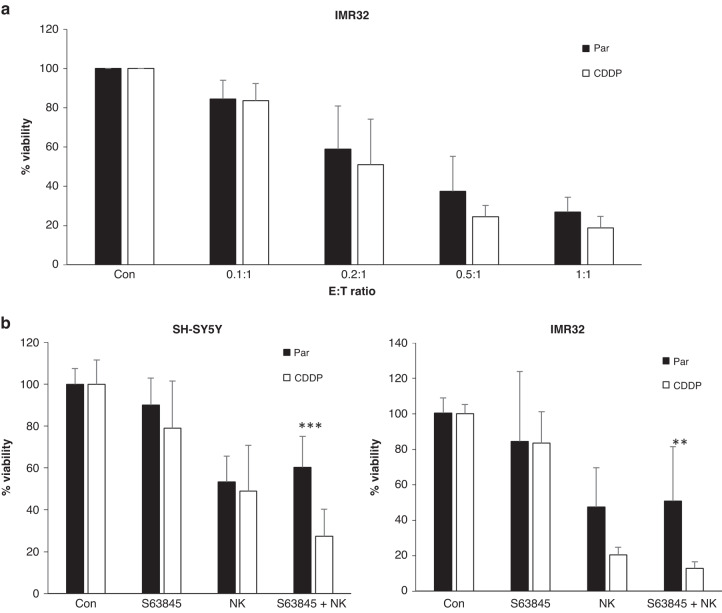
NK cell-based immunotherapy can overcome cisplatin resistance and cooperates with BH3-mimetics. **a** Parental or CDDP IMR32 cells were co-cultured with allogenic IL15-activated NK cells at different effector to target (E:T) ratios for 24 h before washout of NK cells. Cell viability was assessed 4 h after removal of NK cells by CTG assay. **b** SH-SY5Y or IMR32 cells were grown in spheroids for 3 days before treatment with S63845 (1 μM) for 4 h followed by addition of IL15-activated allogenic NK cells at an E:T ratio of 0.2:1 for additional 24 h. Analysis of viability was performed using CTG assay at 72 h after addition of the NK cells. Data shown are mean + SD (*n* = 3), **p < 0.01, ***p < 0.001.

Next, we aimed to extend our study beyond the investigations of drug combinations into the field of cellular immunotherapy. Since we and others [21, 22] have recently shown that BH3-mimetics and in particular inhibitors of MCL1 or BCL-X_L_ may increase the cytotoxicity of NK cells, we asked whether NK cells may be able to combat chemoresistance in neuroblastoma. To this end, we compared the cytotoxicity of IL15-activated allogenic NK cells in parental and CDDP cells. Comparison of different effector to target (E:T) ratios demonstrated that NK cells are able to attack CDDP cells at comparable levels to parental cells (Fig. 6a). Of note, only low E:T ratios of <1:1 were required to efficiently kill both parental and CDDP cells, highlighting the potential of cellular immunotherapy in tackling chemoresistance. To address whether combinations of BH3-mimetics and NK cells may be beneficial, we combined different BH3-mimetics with NK cells before assessing cellular viability. In both IMR32 and SH-SY5Y cells, the combination of S63845 and NK cells was able to efficiently eliminate CDDP and parental cells (Fig. 6b). Taken together, these experiments provide a first and exciting indication that the combination of MCL1 inhibition together with NK cell-based immunotherapy may be highly promising for the treatment of chemoresistant neuroblastoma.

## Discussion

The apoptosis-inducing potential of the BH3-mimetic ABT199/venetoclax is transforming the treatment of adult patients with leukaemia. Early clinical trials indicate that also paediatric patients may benefit from treatment with BH3-mimetics, and clinical trials in children with high-risk acute myeloid leukaemia (AML) or myelodysplastic syndrome (MDS) have demonstrated a favourable safety profile as well as clinical activity of venetoclax [31, 32]. The use of venetoclax in children is currently being further investigated in multiple phase 1 clinical trials in either leukaemia or relapsed/refractory neuroblastoma (NCT04029688 and NCT03236857) [33]. The treatment with BH3-mimetics targeting BCL2 or BCL-X_L_ also appears to be well tolerated in paediatric patients, and the combination of venetoclax with low doses of ABT263/navitoclax has been tested in paediatric acute lymphoblastic leukaemia (ALL) patients with highly promising results [34]. The clinical development of BH3-mimetics targeting MCL1 is not as advanced as the development of BCL2 inhibitors. Although several compounds are currently being investigated in adult patients, including S64315 and AZD-5991 (NCT02979366), safety concerns are slowing down the clinical development of these inhibitors, e.g. in NCT03797261.

Our results indicate that BH3-mimetics may have highly different activity in the treatment of naïve versus relapsed and chemotherapy-resistant cancer. Navitoclax has been investigated as therapeutic option in cisplatin-resistant ovarian cancer, but phase 2 clinical trials indicated poor response rates in a heavily pre-treated patient group [35]. While our study focussed on neuroblastoma, previous studies indicate that a similar shift towards MCL1 dependency may be observed in other cancer types as well. To this end, several reports have shown an upregulation of MCL1 in chemoresistant cancer [36, 37]. This may be mediated by gene amplification of MCL1 in subpopulations of cancer cells that encounter a survival benefit during chemotherapy [36]. Alternatively, MCL1 protein has been shown to be stabilised by upregulation or activation of its deubiquitinase during chemotherapy [38, 39]. As a CAP-dependent transcript, MCL1 expression may also be influenced via translational regulation [40], which could contribute to higher MCL1 expression in chemoresistant cancer. A non-canonical function of MCL1 in promoting chemoresistance has been described involving nuclear translocation and a BH3-binding domain-independent function of MCL1 [41].

While a prominent role of MCL1 in chemoresistant cancer has been described in multiple studies, the loss of sensitivity to BCL2/BCL-X_L_ inhibitors observed here may be more controversial and potentially limited to neuroblastoma. Several studies have described BCL2 as upregulated in chemoresistant cells, and a recent screen for genes important in chemoresistant AML identified BCL2 as prominent gene [42]. Similarly, paclitaxel-resistant triple-negative breast cancer cells were more sensitive to ABT263 than their parental counterparts indicating that, at least in some tumour types, also BCL2 and BCL-X_L_ may be highly promising targets. In cisplatin-resistant neuroblastoma, however, MCL1 was by far the most promising anti-apoptotic target, with sensitivity to BCL2 and BCL-X_L_ inhibitors lost upon chemoresistance. The underlying mechanism for this shift in sensitivity is currently unknown, but may involve a shift in binding of BIM from BCL2 to MCL1, coinciding with increased MCL1 and reduced BCL2 expression. The observation that NOXA and MCL1 both appear to be upregulated in the CDDP cells, may indicate that also the complex formation between NOXA and MCL1 is increased, and that this complex may lead to more stable NOXA protein. Overall, these altered expression patterns may lead to a higher addiction of the CDDP cells to MCL1. However, the NLF CDDP cells also display increased MCL1 protein expression without an increase in MCL1 inhibitor sensitivity, indicating that other factors may contribute as well.

Of note, the apoptosis resistance observed in the CDDP cells is entirely mediated by the BCL2 family, since the simultaneous inhibition of all three main anti-apoptotic BCL2 proteins (BCL2, BCL-X_L_ and MCL1) is sufficient to kill the CDDP cells. These data highlight that neither drug efflux transporters nor the upregulation of survival signalling is immediately responsible for the apoptosis resistance observed in the CDDP cells. Instead, the inhibition of the anti-apoptotic BCL2 proteins may represent an Achilles heel of chemoresistant cancer, and hence the application of BH3-mimetics in chemoresistant disease appears to be highly promising.

To identify novel ways to apply BH3-mimetics in chemoresistant cancer, we performed a drug screen with FDA-approved anti-cancer drugs. This screen identified several drugs previously shown to increase the activity of ABT263 and related BCL2/BCL-X_L_ inhibitors like MEK inhibitors or CDK inhibitors [43, 44]. Mechanistically, these compounds are known to reduce MCL1 expression, thus providing an explanation for the synergy with ABT-263. In addition to these kinase inhibitors, we identified the natural compounds triptonide and triptolide as sensitisers for ABT263. Triptolide has previously been described as synergistic with ABT199 in AML cells by downregulating MCL1 [45]. Interestingly, our screen identified no compounds that displayed convincing synergy with the MCL1 inhibitor S63845. However, since the screen was performed only in one cell line, this may not be representative for all resistant neuroblastoma cases. Given the safety concerns identified in clinical trials with MCL1 inhibitors, it appears particularly important to identify strategies in which low doses of an MCL1 inhibitor may be sufficient to sensitise tumour cells without unwanted toxicities.

While no pharmaceutical drugs were discovered that amplified the apoptosis-inducing capacity of S63845, we identified cellular immunotherapy based on NK cells as highly promising option for the treatment of relapsed neuroblastoma. Thereby, we concentrated on NK cells rather than cytotoxic T-cells, since NK cells are safely applicable in an allogenic and more cost-effective setting. Both NK and T-cell activity may be tailored and enhanced by the expression of Chimeric Antigen Receptor (CAR) constructs. To this end, several promising targets have been identified in neuroblastoma, including DNAM-1, GD2 and B7-H3 [46–49]. Here, we only investigated allogenic PBMC-derived NK cells that were activated ex vivo using IL-15, and we anticipate that the activity of NK cells may be further increased by the introduction of a CAR construct. Our findings that NK cells are equally effective in both parental and in CDDP cells and that only low E:T ratios of 0.2:1 are sufficient to kill neuroblastoma cells, highlight the potential of NK cell-based immunotherapy for the treatment of relapsed neuroblastoma. The efficacy of NK cells in killing neuroblastoma cells has previously been shown, demonstrating that NK cells harbour an important graft-versus-tumour activity [50]. Of note, in patients with neuroblastoma, chemotherapy may lead to a NK cytopenia, and NK cell infusions may counteract the loss of NK cells and increase NK cell cytotoxicity [51].

Here, we show that the activity of NK cells may further be increased by the addition of BH3-mimetics, in particular the MCL1 inhibitor S63845. These observations highlight that BH3-mimetics may sensitise tumour cells for immune attack, as we and others have recently shown for other tumour entities [21, 22]. Mechanistically, treatment with BH3-mimetics may facilitate killing via the mitochondrial pathway, and thus allow NK cells to effectively activate apoptosis in the targeted tumour cell. Importantly, the synergistic activity of NK cells together with the MCL1 inhibitor S63845 was particularly striking in chemoresistant CDDP cells, thus outlining a novel combination therapy able to overcome chemoresistance.

In conclusion, we discovered a prominent shift in the sensitivity to BH3-mimetics upon the occurrence of chemoresistance, demonstrating that in chemoresistant neuroblastoma MCL1 is a highly promising therapeutic target. The effect of selective BH3-mimetics targeting MCL1 may further be increased by combination with cellular immunotherapy, thus opening up the possibilities of a safe and non-toxic combination for the treatment of relapsed neuroblastoma.

### Supplementary information

Supplementary Material

## Data Availability

The datasets generated during the current study are available from the corresponding author on reasonable request.
