# Supplementary material for: Increased MCL1 dependency leads to new applications of BH3-mimetics in drug-resistant neuroblastoma
Source: Br J Cancer. 2023 Sep 19;129(10):1667–78. doi: 10.1038/s41416-023-02430-8 (PMC10646009; doi:10.1038/s41416-023-02430-8)
Supplement: Supplementary file 1 — Supplementary Material [file 41416_2023_2430_MOESM1_ESM.pdf]

Supplementary Figure 1

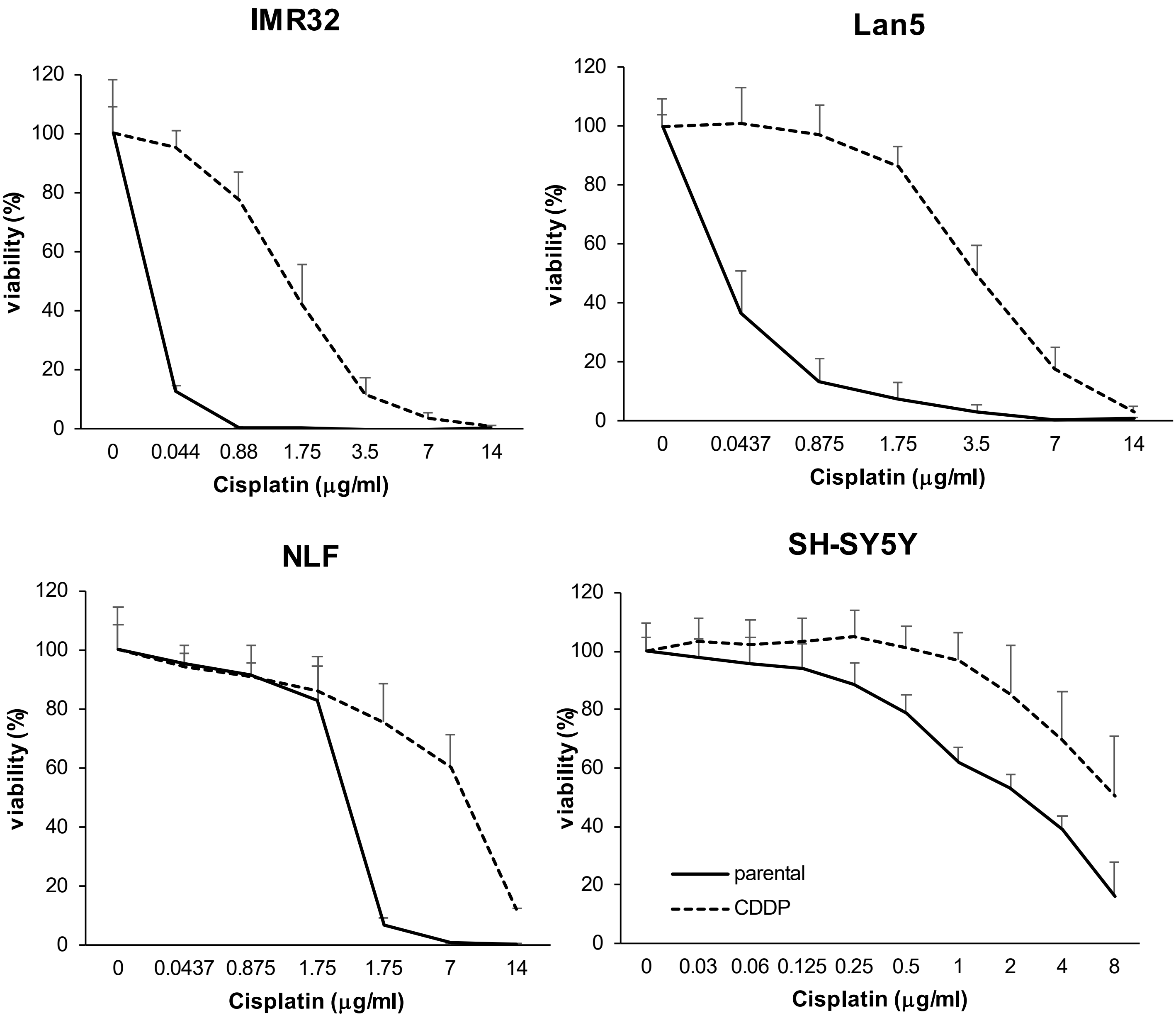

Supplementary Figure 2

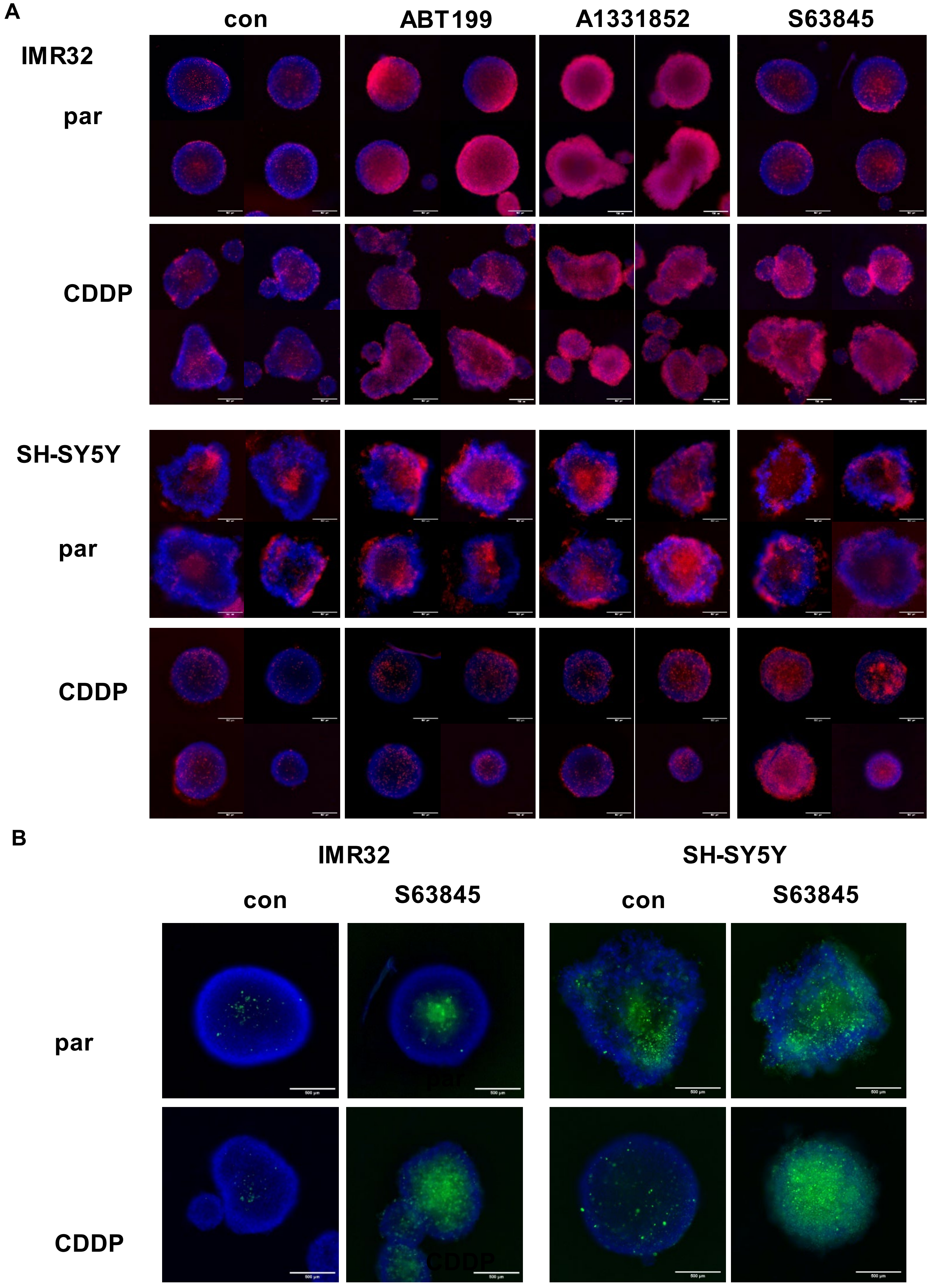

Supplementary Figure 3

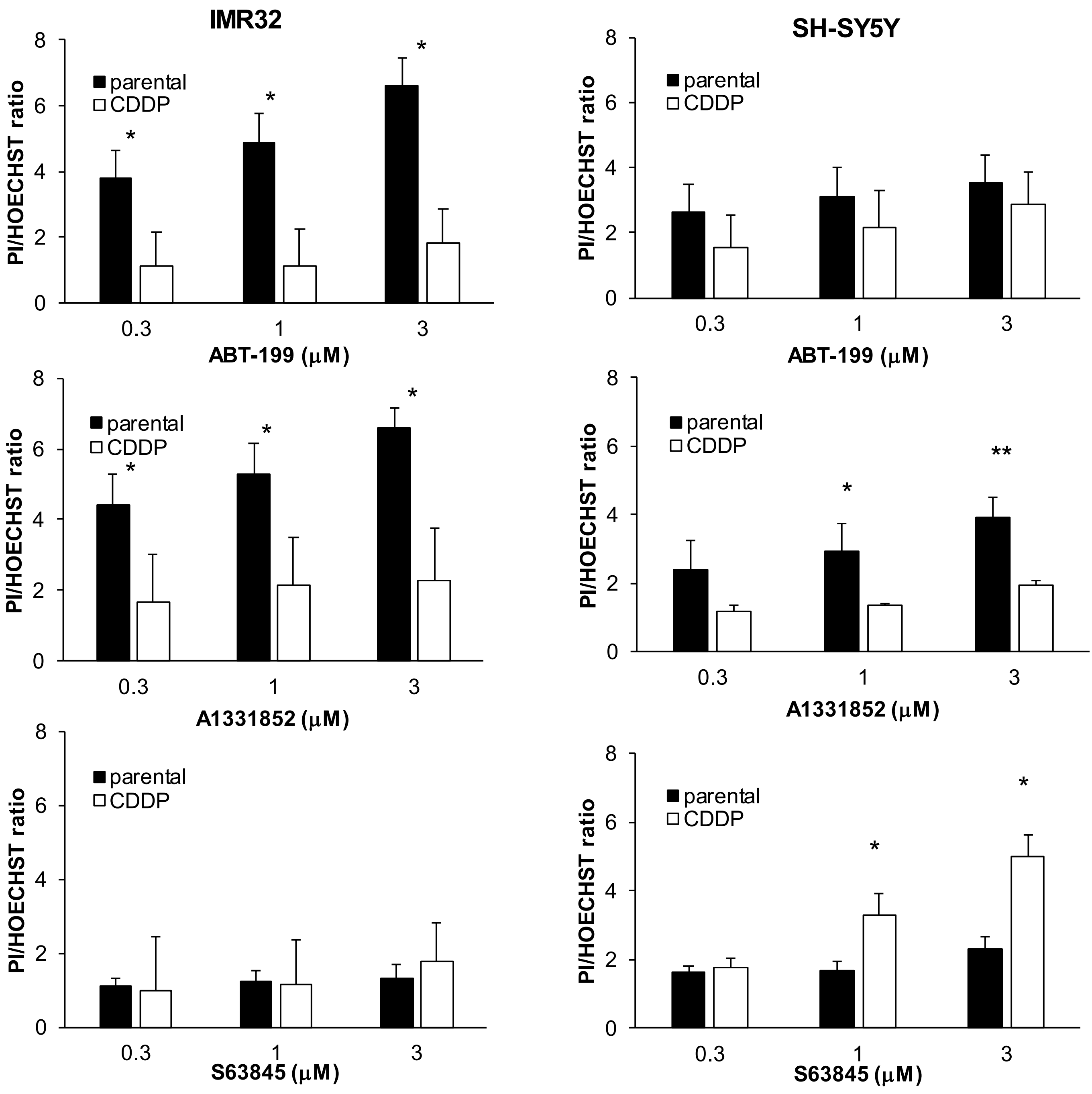

Supplementary Figure 4

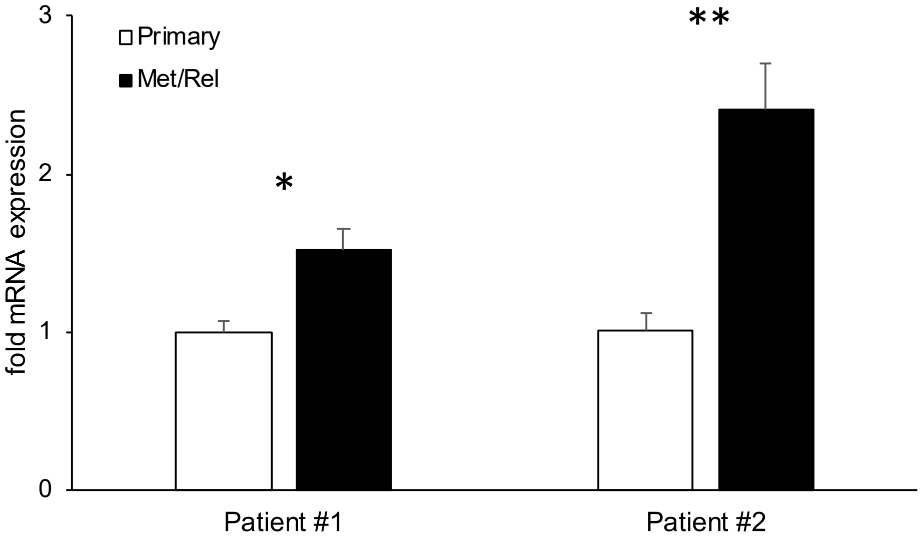

Supplementary Figure 5

A

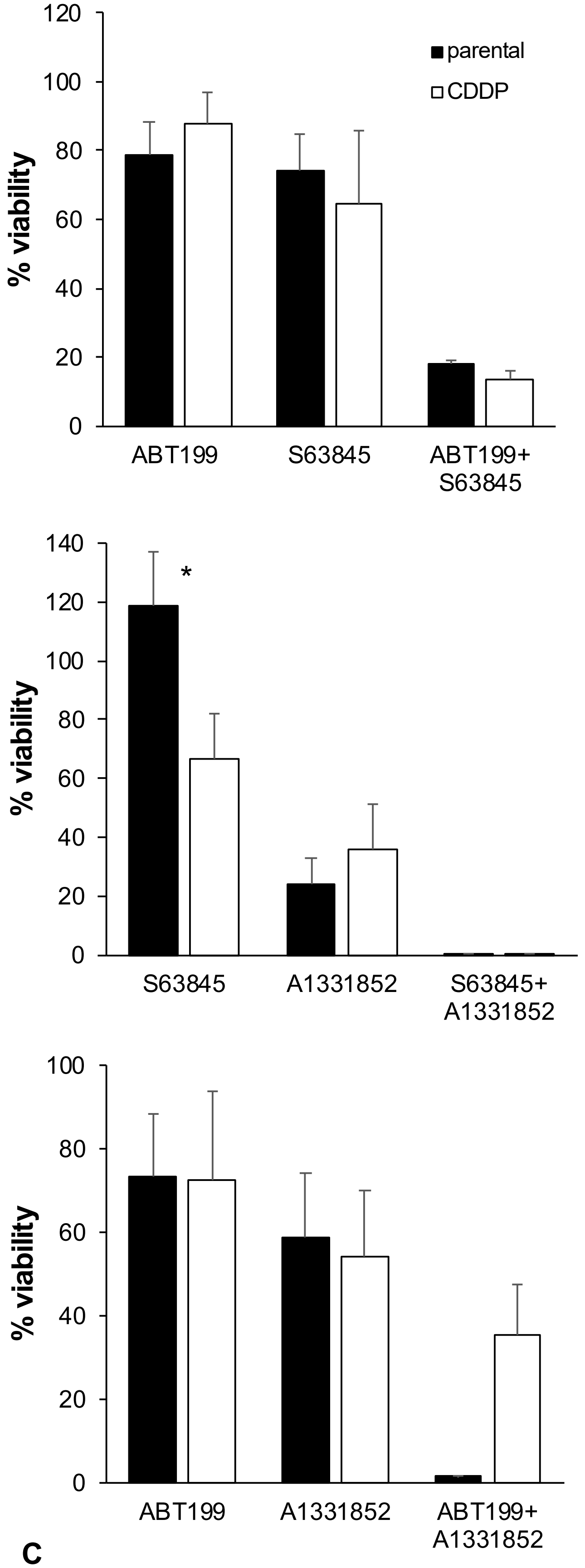

B

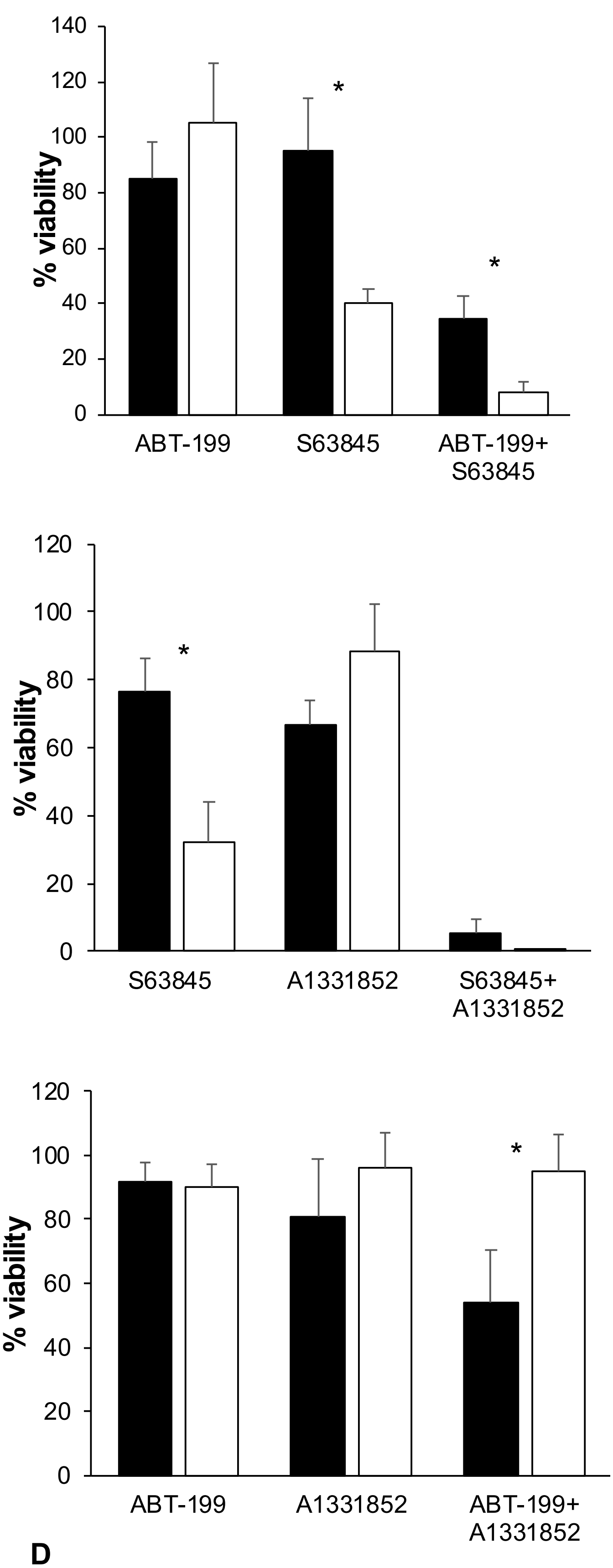

C

IMR-32	parental	CDDP
ABT199 + S63845	49,5	50
S63845 + A1331852	34,7	36,3
ABT199 + A1331852	26,2	19,7

D

SH-SY5Y	parental	CDDP
ABT199 + S63845	66,7	38,8
S63845 + A1331852	56,9	38
ABT199 + A1331852	18,9	-0,9

Supplementary Figure 6

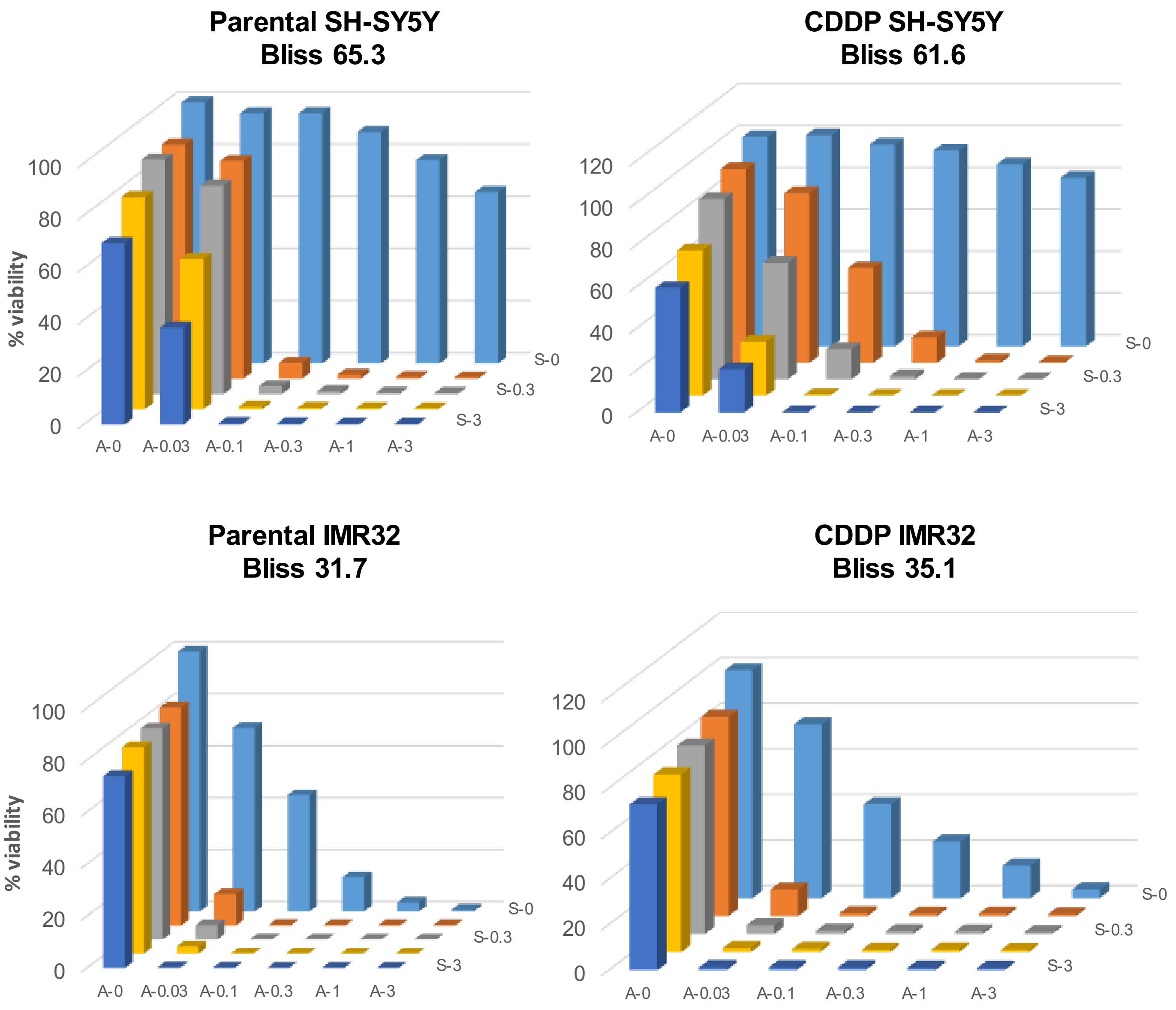

Supplementary Figure 7

Chemo	BH3 mim	Lan5-CDDP	IMR32-CDDP
Cisplatin	ABT199	8.8	6.7
Etoposide	ABT199	6.8	8.7
Doxorubicin	ABT199	-0.4	10.5
Cisplatin	A1331852	5.2	19.8
Etoposide	A1331852	2.1	11.6
Doxorubicin	A1331852	2.2	10.9
Cisplatin	S63845	4.4	14.2
Etoposide	S63845	7.5	13.4
Doxorubicin	S63845	8.6	9.6

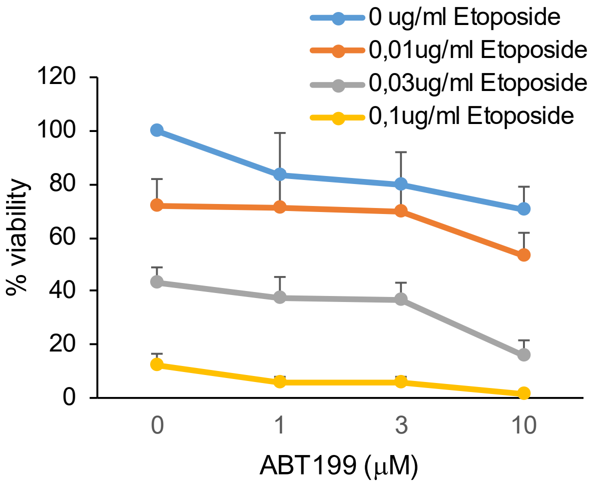

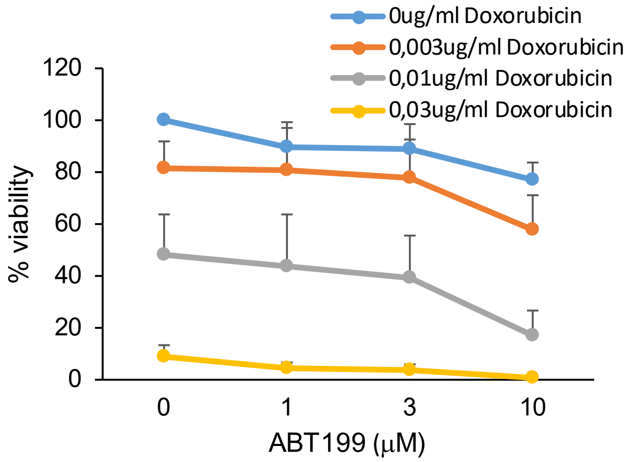

Supplementary Figure 8

CHLA-20

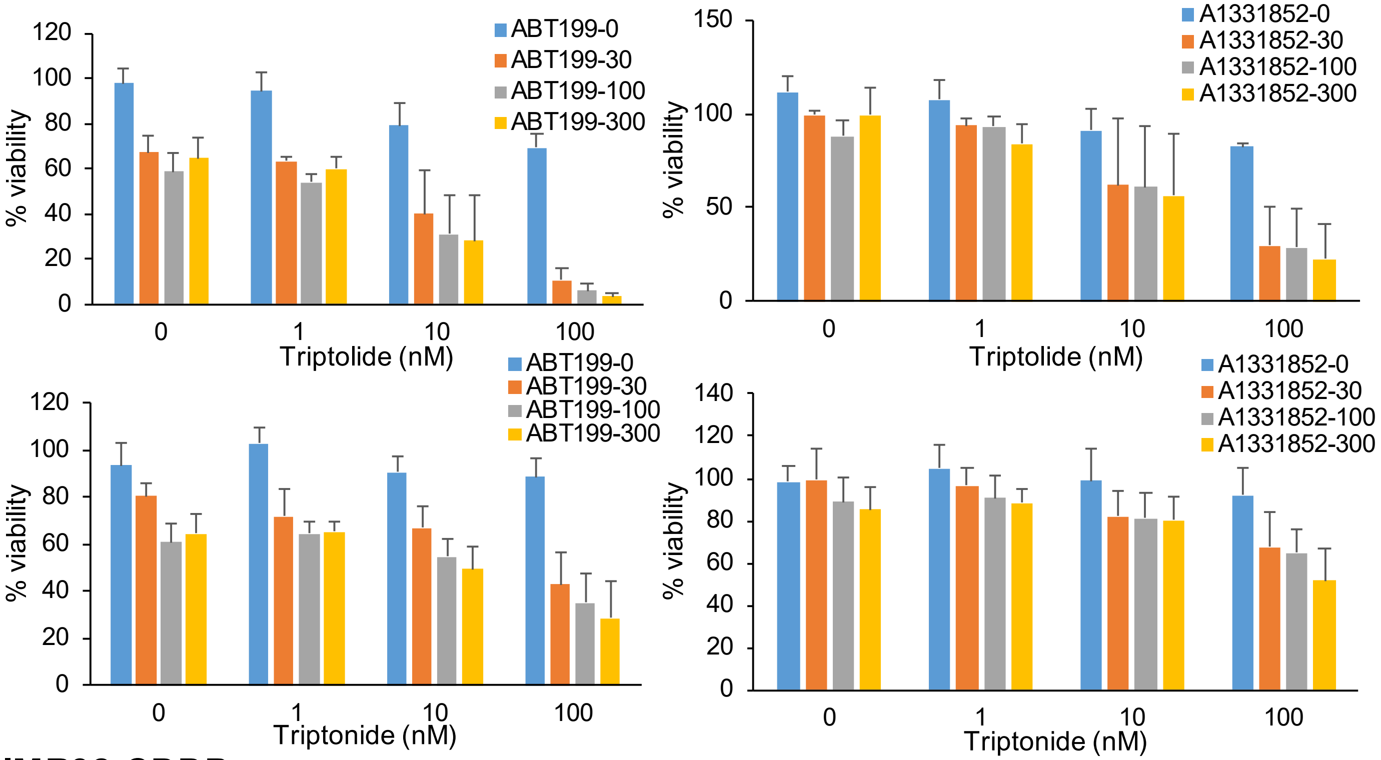

IMR32-CDDP

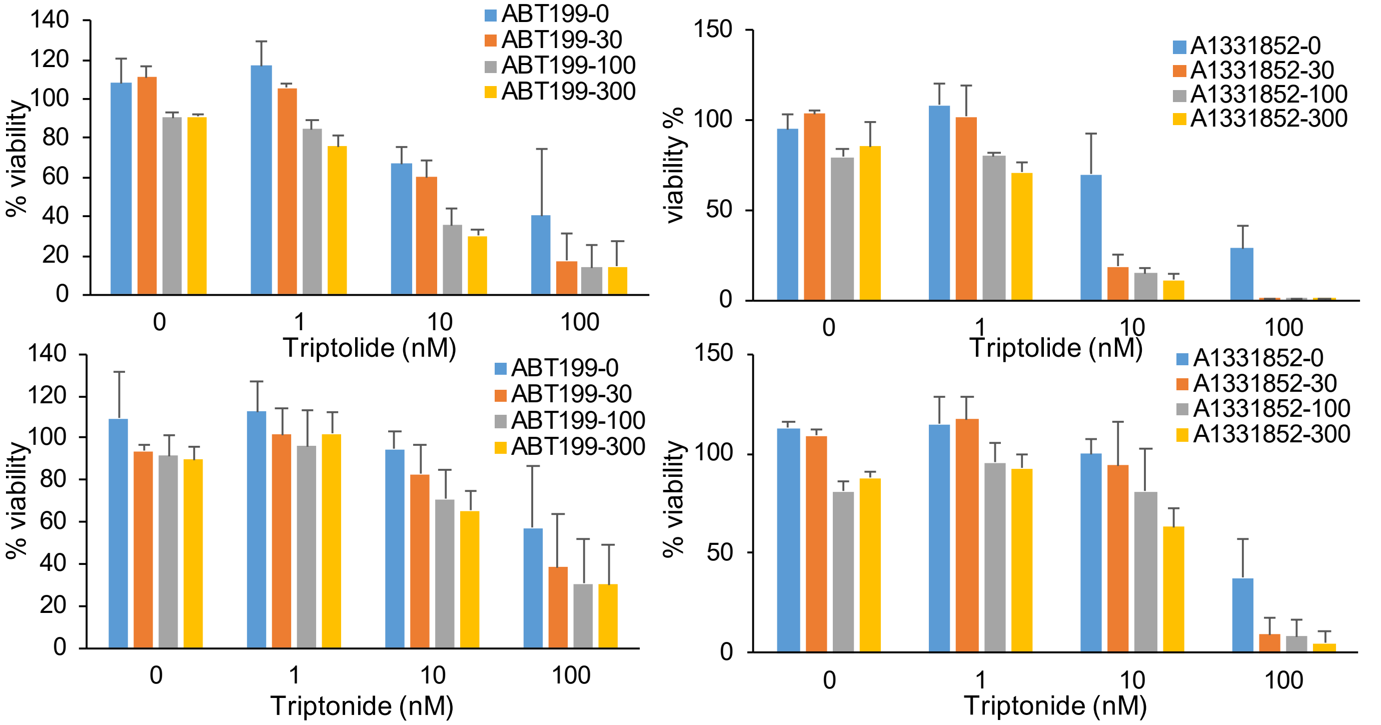

		CHLA20	IMR32-CDDP
**ABT263**	**Triptolide**	21.9	13.2
**ABT199**	**Triptolide**	27	11.7
**A1331852**	**Triptolide**	21.8	23
**ABT263**	**Triptonide**	6.2	6.2
**ABT199**	**Triptonide**	12.6	5.1
**A1331852**	**Triptonide**	6.9	3

Supplementary Figure Legends

Supplementary Figure 1. Cisplatin response in parental and CDDP cells.

IMR-32, Lan5, NLF or SH-SY5Y neuroblastoma cell lines were treated with different concentrations of cisplatin for 72 hours before analysis of viability using CTG assay. The response of parental cells (solid lines) was compared to the response of CDDP-resistant cells (dashed lines). Data shown are mean + standard deviation (SD) with n=3-4.

Supplementary Figure 2. Altered response to BH3-mimetics in neuroblastoma spheroids.

Parental or CDDP-resistant IMR32 or SH-SY5Y cells were cultured as tumour spheroids for 3 days before treatment with different BH3-mimetics (1 μM) for 48 hours. A) Tumour cell killing was assessed by staining with HOECHST (blue) and propidium iodide (PI, red) using microscopy. Four representative spheroids are shown for each condition. B) IMR32 or SH-SY5Y spheroids were treated with S63845 (1 μM) before analysis of caspase activity (green) and staining with HOECHST (blue). Representative images of three independent experiments each performed in triplicate are shown. Scale bar equals 500 µm. For quantification of cell death see Supplementary Figure 3.

Supplementary Figure 3. Quantification of cell death in 3D spheroids treated with BH3-mimetics.

IMR-32 and SH-SY5Y spheroids were grown for 4 days before treatment with different concentrations of BH3-mimetics for 24 hours and analysis of cell death using staining with PI and HOECHST followed by plate-based microscopy. Examples of the spheroids are shown in Supplementary Figure 2. Cell death was quantified as the ratio of PI- versus HOECHST-stained cells. Data shown are mean + SD (n=4), *: $p<0.05$,**: $p<0.01$.

Supplementary Figure 4. Expression of MCL1 mRNA in neuroblastoma tissues

RNA was isolated from neuroblastoma tumour tissues obtained from two individual patients at initial diagnosis (primary) or relapse (metastasis/relapse). Expression of MCL1 mRNA was analysed by qRT-PCR and normalised to housekeeping genes. Data shown are fold changes comparing metastatic/relapse samples to primary tumours (mean +SD of technical repeats (n=3) with *: $p<0.05$,**: $p<0.01$).

Supplementary Figure 5. BH3-mimetics act synergistically in cisplatin-resistant neuroblastoma cells.

Parental or CDDP IMR32 (A) or SH-SY5Y (B) cells were treated with combinations of BH3-mimetics (1 μM) for 72 hours before analysis of viability using CTG assay. Data shown are mean + SD (n=3), *: $p<0.05$,**: $p<0.01$. C and D) To quantify the synergy between the different BH3-mimetics, the Bliss score was calculated for different concentrations (0.1 – 10 μM) of BH3-mimetics.

Supplementary Figure 6. ABT263 and S63845 act highly synergistically in cisplatin-resistant neuroblastoma cells.

Parental or CDDP IMR-32 or SH-SY5Y cells were treated with different concentrations of ABT263 (A-0 to A-3) and S63845 (S-0 to S-3) for 72 hours before analysis of viability using CTG assay. Data shown are mean + SD (n=3), *: $p<0.05$,**: $p<0.01$. To quantify the synergy between the BH3-mimetics, the Bliss score sum was calculated and is displayed in the Figure.

Supplementary Figure 7. BH3-mimetics combined with chemotherapeutic drugs in cisplatin-resistant neuroblastoma cells.

CDDP IMR-32 or Lan5 cells were treated with different concentrations of selective BH3-mimetics and the chemotherapeutic drugs cisplatin, etoposide or doxorubicine for 72 hours before analysis of viability using CTG assay. To quantify the synergy between the different

combinations, the Bliss score sum was calculated and is displayed in the table. An example of a dose response for the IMR32-CDDP cells treated with ABT199 and etoposide (left) or doxorubicin (right) is shown. Data shown are mean + SD (n=3).

Supplementary Figure 8. BH3-mimetics combined with natural compounds in cisplatin-resistant neuroblastoma cells.

CHLA-20 or IMR-32-CDDP cells were treated with different concentrations of selective BH3-mimetics and the natural compounds triptolide (upper panels) or triptonide (lower panels) for 72 hours before analysis of viability using CTG assay. To quantify the synergy between the different combinations, the Bliss score sum was calculated and displayed in the table. Data shown are mean + SD (n=3).
